# Colombo Twin and Singleton Study (CoTASS): A description of a population based twin study of mental disorders in Sri Lanka

**DOI:** 10.1186/1471-244X-8-49

**Published:** 2008-06-27

**Authors:** Sisira H Siribaddana, Harriet A Ball, Suwin N Hewage, Nick Glozier, Yulia Kovas, DARK Dayaratne, Athula Sumathipala, Peter McGuffin, Matthew Hotopf

**Affiliations:** 1 Sri Lanka Twin Registry, Institute of Research and Development, Battaramulla, Sri Lanka; 2 Social Genetic and Developmental Psychiatry Centre, Institute of Psychiatry, London, UK; 3 The George Institute for International Health, University of Sydney, Sydney, Australia; 4 Section of Epidemiology, Institute of Psychiatry, Kings College, University of London, London, UK; 5 Department of Psychological Medicine, Institute of Psychiatry, Kings College, London, UK

## Abstract

**Background:**

The Sri Lankan twin registry is one of the first to be established in a developing country, and its design has ensured sampling from a wide range of environmental conditions. It thus has great potential to examine environmental and genetic influences on diverse phenotypes, including psychiatric disorders, in the context of a diversity of environmental exposures, which may not have been fully explored in previous twin studies in developed countries. This paper presents the rationale for the study, describes its context, and the methods for twin ascertainment and data collection.

**Methods:**

A population-based twin register was established in the Colombo district of Sri Lanka using infrastructure designed to periodically update the electoral register. We invited a subsample from this register to participate in the project on common mental disorders, using random ascertainment. A separate non-twin sample was randomly selected from the geographical areas where twins were found. Home interviewers collected diagnostic information on common mental disorders, as well as environmental exposures including life events, socio-economic conditions, and the impact of the civil war and the Tsunami of 2004.

**Results:**

We identified 19,302 individuals in the creation of the population based twin register. We randomly selected a subsample, of whom 4,387 were eligible to participate and 4,024 agreed to be interviewed (including data on 1,954 complete pairs of twins and 5 sets of triplets). Those who refused consent had a similar mean age and sex ratio to those who were interviewed. We invited 2,485 singletons to participate and 2,019 were interviewed.

**Conclusion:**

Initial exploration of the data suggests the samples are very representative of the Colombo district of Sri Lanka, so we have created a unique resource for understanding the influences on mental disorders in developing countries, and to compare to the influences found in developed countries.

## Background

Twin studies can provide insights into the heritability of psychiatric disorders, and the extent to which within-population variation in psychiatric disorders is influenced by the environment. They can further describe genetic and environmental contributions to overlap between disorders, and provide information on likely gene environment interactions. Previous twin studies have found that 30–70% of liability to major depression is influenced by genetics [1,2], as is roughly 40% of psychosomatic symptoms [3], and 50% of alcoholism [4]. Findings of environmental influences that are shared between twins, including family-wide influences, have been of modest magnitude at most, and often absent, in assessments of common mental disorders in adult twins. Heritabilities of over 70% have rarely been reported, leaving room for substantial influences from a combination of environmental factors that are not shared across twins, and for measurement error.

Heritability is an estimate of the causes of variability of a phenotype within a given population. However, a high heritability does not rule out important environmental exposures if these do not vary within the population. For example, if there are important environmental influences on a phenotype, but these exposures do not vary within the specific population, then the phenotype would have a high estimated heritability. Examining populations in diverse environmental settings may increase our understanding of causal influences that are not detectable in populations that are environmentally more homogeneous. For example, the wider range of standards of living experienced in Sri Lanka compared to the West may lead to a greater magnitude of environmental influence on mental disorders. The Sri Lankan environmental context will also allow testing of measurement invariance, for phenotypes that have previously been measured in developed countries.

Past twin studies may not have taken full account of the distribution of environmental risk factors. Most have been confined to developed Western countries, which do not encompass the full range and potency/severity of environmental exposures seen throughout the world [5,6]. Some have relied on volunteer samples rather than being truly population-based, and such ascertainment may bias or restrict the variation of socio-economic influences sampled [7] (e.g. underrepresentation of those with few educational qualifications [8]). It is possible that the experience of being a twin affects the exposures and phenotypes under study [9,10]. Twins may differ from non-twins for many reasons, especially those twins included in genetic analyses, since the twin pair must be intact: in other words, both twins must still be alive and willing to participate. The surviving twin pairs may be unrepresentative of the general population, especially if being a twin is associated with a higher infant mortality rate, which may be particularly germane to developing countries. Finally, twin studies estimate genetic and environmental effects indirectly, by comparing the similarity of MZ pairs to the similarity of DZ pairs. They can thus provide estimates of environmental influences without having to measure the environment, but the precise nature of these environmental influences is unknown unless the exposures are directly measured [11].

The twin study we describe aims to overcome these shortcomings. Firstly, it is based in Sri Lanka, a developing country with a per capita gross national income of approximately $1,160, and wide disparities of wealth and environmental exposures. Secondly, we aimed to identify all twins within a defined population, and invite a random sample to participate in the study. Thirdly, we directly measured a range of environmental exposures, both those comparable to populations in developed countries, and exposures specific to this population. Finally, we identified and sampled non-twins in a similar manner, in order to examine how representative exposures and outcomes reported by twins are to the general population.

Sri Lanka has a high life expectancy (74 years), low infant mortality rate (12/1000/year) and high literacy rates (91%) despite its status as a developing country [12]. In contrast to these favourable quality of life indices, the country has a high suicide rate (up to 40/100000/year) [13], and a high prevalence of alcohol dependence (29/1000) [14]. Consumption of alcohol and deaths from alcoholic liver disease have both risen sharply in the past 20 years and a government document stated "it is no secret that Sri Lanka is listed as one of the countries with (the) highest alcoholism levels" [15].

The country has been affected by a prolonged civil war with hostilities in the North and East between the government and Tamil separatist forces. Whilst the Colombo district has been relatively spared from this unrest, a proportion of the residents will have had direct experience. Sri Lanka was also affected by the Tsunami of 26^th ^December 2004 with approximately 40,000 deaths. These deaths were predominantly in the South and East of the island, but the Colombo district was affected with the loss of 56 lives. 3,397 homes were damaged to the extent that they could not be used, and partial damage of a further 2,948 homes [16].

The aim of the present paper is to describe the methods we used to conduct this study, and to provide baseline results on the nature of the samples identified.

## Methods

### Setting

The twin register is based in Colombo, one of the 25 districts of Sri Lanka. Colombo District includes the capital city, its suburbs and surrounding rural areas. The population of this district is 2.23 m according to the 2001 census [17]. There are three main ethnic groups: the majority Sinhalese (76.6% in Colombo), who speak Sinhala are predominantly Buddhist; the Sri Lankan Tamils (11.0%) who are a majority group in the North and East, speak Tamil (although most in Colombo speak Sinhala as well) and are predominantly Hindu; and the Moors (9.2%) who are descended from Arabic traders, are predominantly Muslim and speak Tamil and Sinhala.

### Participants

#### Identification and sampling of twins

We attempted to identify twins from birth records and key informants. Whilst this method was successful for young twins, it was not efficient to identify older ones [18]. Instead, we opted for a modified door-to-door survey, making use of Sri Lanka's annual update of the electoral register. This is administered by civil servants (called 'Grama Nildhari'), who are each responsible for one of Colombo district's 557 Grama Niladhari Divisions (GND, the smallest administrative unit, with approximately 4,000 people in each). A form that inquired about any twins residing in the household, or any twins known to the informant, was distributed and collected alongside the annual census visits. In some districts, electoral officers completed this work. We screened the information to exclude duplicate reporting and pairs where both twins resided outside of Colombo district. We incorporated the twins from the island-wide volunteer database [19] and feasibility studies [20] into this Colombo database. We used random numbers to sample 6,600 twins aged 15 years or older from this database. Twins were excluded if the individuals said they were not twins; one or both of the pair had died or gone abroad; or there were no twins at the given address.

#### Sampling of non-twins

During the course of the twin study, we also identified all households in Colombo in which no twins were residing. This household data was organised into GNDs. Having performed the twin sampling, we identified the GND from which each selected twin came. The non-twins were sampled from the lists specific to each GND in a ratio of 1:1 (twin pair: non-twin individual). Households were selected using random numbers such that each household in the GND had an equal chance of selection. Letters were sent to the selected households explaining about the study and the twin registry field workers visited the house to determine how many individuals over 15 years were residing there. One research participant from each selected household was selected at random from this list. The consequence of this approach is that the non-twin sample was selected from the same geographical area as the twin sample, but no other socio-economic variables have been used to match the two groups.

### Assessment

#### Training and supervision of fieldworkers, and quality control

A group of 10 individuals (mostly doctors with no mental health experience) were extensively trained in the Composite International Diagnostic Interview by NG, a CIDI approved trainer [21]. These individuals were trained not only in the use of the measure but also to train field workers. A further 34 field workers were recruited. The field workers had A-level or graduate level education, but no experience in healthcare. The field workers completed a 14-day intensive course, which involved different methods including role play and mock interviews. Apart from being trained in the use of CIDI, and other measures described below, the field workers were instructed on multiple relevant skills (such as interview techniques; emphasising the importance of informed consent; allowing participants to refuse consent) and knowledge (general information about the purpose of the study and the nature of mental disorders). After this, field workers performed pilot interviews among their friends and relatives. There was a further 3 day training session where field workers were asked to recount difficulties in the use of the measure, and perform mock interviews and role play. One month after the start of the main data collection, a further 2 day training session was conducted in order to detect difficulties and consolidate knowledge. During the course of the study field workers were given weekly group supervision where any difficulties in the use of the measures was discussed. Each interview was checked by a trainer in the CIDI who would provide feedback to individuals if any errors or problems in the interview were detected. Project managers (SS and SH) performed random checks by contacting participants to ensure that the interviews had been conducted. Further queries on CIDI questions were referred to NG during the study. Where practical, a different interviewer was used for each member of the twin pair.

#### Translation and adaptation of measures

We sent the measures to a total of 13 bilingual twins (contacted from the registry) and other Sri Lankans fluent in English and Sinhala. Each measure (or in some cases subcomponent of measures) was translated at least twice independently. The translations were then reviewed in group meetings consisting of 7 professionals (6 doctors and 1 health service researcher, all with a background relevant to the measures – e.g. mental health) over a period of 4 months. A scholar in Sinhala also checked the translation. The adaptation was not a direct, literal translation, but aimed to find forms of words in Sinhala that best described the concepts of interest and where the questions when translated seemed cumbersome, they might be broken down into two component items for clarity [22]. Some measures were adapted for local use, such as the card in the CIDI to show alcohol consumption, which included local drinks such as toddy and illicitly distilled spirits. The interviews were then trialled by multiple volunteers recruited from field workers and four individuals with no connection to the study, in order to confirm that lay people could understand it.

### Procedure

All twins were sent information about the registry and the present study. Participants who were randomly selected were then sent a more detailed letter providing information and explaining their freedom to refuse consent if they did not want to participate. They were informed that interviews could take place in their own home or at the research centre. Twins residing in more rural GNDs were again sent a letter with a stamped addressed envelope for them to provide information about how researchers could reach their home. A date was then set for an interview by phoning or a visit. A full information sheet was given and the participants were encouraged to read it carefully and if necessary, time was given to discuss with close ones before consent was given. Another appointment was made after a week to obtain written informed consent with separate consent for a buccal smear for zygosity testing on same sex twins. Field workers completed the interview (consisting of 88 pages, and lasting 45–150 minutes) and offered a payment of 300 Rupees (approximately £1.50) in compensation for their time, at the end of the interview. Compensatory payment was not mentioned in the information provided prior to the interview. A substantial percentage of the participants refused the payment and instead requested it to be donated back to the research project.

### Measures

#### 1. Phenotypic Data

The subjects were interviewed using the WHO-Composite International Diagnostic Interview [21], for current and lifetime diagnoses. This fully structured interview gives psychiatric diagnoses corresponding to all DSM-IV and ICD-10 psychiatric disorders, including affective, anxiety, somatoform and substance misuse disorders, and posttraumatic stress disorder. It has been widely used in developing countries and can be administered by lay interviewers. We excluded somatoform disorders and limited the substance misuse section to alcohol and nicotine use.

Symptomatic fatigue was measured on the Chalder Fatigue Scale [23], a 13 item questionnaire on fatigue symptoms using a Likert score. Somatic symptoms were measured on the Bradford Somatic Inventory [24], a 21 item scale on common somatic symptoms derived for use in South Asian populations in the UK, and the Short Form-36 Health Survey [25-27] which has been used extensively in health surveys and asks 36 questions on health – we excluded the mental health inventory. A brief (4 item) questionnaire on suicidal ideas was asked [28].

#### 2. Environmental Exposures

Subjects were also questioned on their exposure to potential environmental stressors. We used the Childhood Experience of Care and Abuse Questionnaire (CECA-Q), but excluded questions on sexual abuse as this was thought to be culturally inappropriate in this setting of an epidemiological survey. Life events were ascertained using an 11 item questionnaire (derived from [29]). We constructed a questionnaire covering socio-demographic factors (including marital and occupational status, education, financial hardship, household tenure, building construction and basic facilities including water and toilet), and another questionnaire to assess disruption specifically due to the Tsunami of 26 December 2004, and due to the civil war.

### Development of new measures

Poverty related variables were derived from a recent census and adapted from other studies in South Asia. We performed a focus group of life events considered important in the local context. These were covered largely by a generic life events measure [29]. We identified involvement in the civil war and tsunami as important potential risk factors and created a set of questions that attempted to identify the extent to which the individual had been involved, if at all – this included a hierarchy of exposures, from losing close family members, to having damaged property. We adapted a measure of suicidal ideation from previous research [28].

### Data management

Data were collected in paper format, and were entered into a database using SPSS [30]. We used twin numbers used in the population based twin registry when entering the data and thereby preventing identification by the researchers but maintaining the link between the data and identity of the twins (linked but anonymous data). We entered a subset of the data twice (all data for 200 of the participants) in order to check for data entry errors. Such errors were sufficiently rare (under 1%) that we decided to proceed without double entering the remaining data.

### Ethics and governance

This research was funded by the Wellcome Trust (UK), and was executed as a collaboration between the Institute of Psychiatry, King's College London (IoP), the World Health Organisation's Human Genetics Programme, and the Sri Lankan Twin Registry. The initial pilot work was approved by the IoP Research Ethics Committee and the Research Ethics Committee of the Faculty of Medicine, Colombo. The main research received the necessary approvals from the Research Ethics Committees of the Institute of Psychiatry, World Health Organisation (Geneva) and Sri Jayewardenepura University of Sri Lanka. This research led to the establishment of ethical guidelines appropriate to twin research in developing countries.

### Zygosity

We assessed zygosity using a questionnaire scored out of 10, asked to both twins in a pair [31]. This questionnaire has a validated cut-off score to indicate zygosity from both twins' reports (score of 14 or greater out of 20 indicates DZ) [32]. DNA testing from buccal smears will be performed [33] at a later date in order to improve validity and classify unknown zygosity.

### Statistical Analysis

Statistical analysis shown here were conducted in STATA [34] and Statistical Package for Social Sciences (SPSS) [30]. Structural equation modelling of the data in order to examine the relative influences of genetic and environmental factors will be performed in the Mx [35] and Mplus [36] packages.

## Results

### Twin Study

A previous paper [37] describes the results of the household survey to identify twins. In all, 66% of the 510,835 survey forms distributed were returned. Among the 13 districts of Colombo surveyed, the return rate was lowest in the most densely populated areas (Colombo (49.6%) and Thimbirigasyaye (32.6%)) and highest in the semi-urban areas to the East of the capital (Padukka (82.8%) and Hanwella (88.0%)).

**Figure 1:**
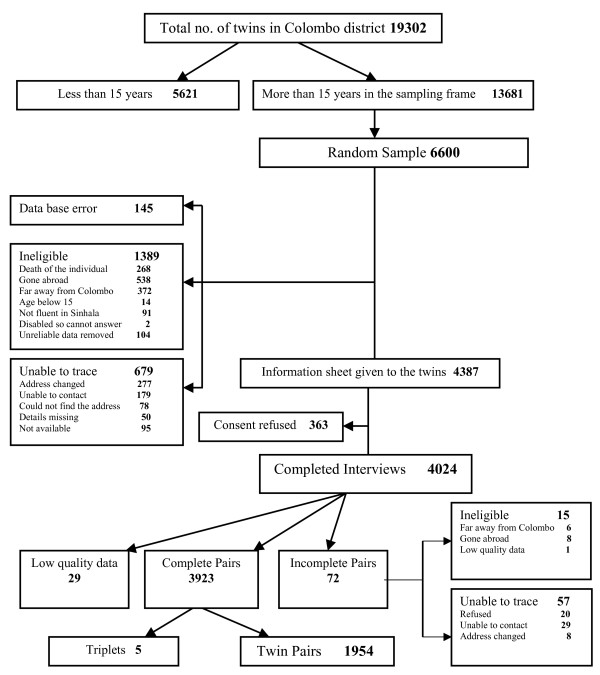
Flow chart of sampling process.

The population of twins identified in Colombo numbered 19,302. Of these 5,621 were excluded from the sampling frame because they were under 15 years. Of 13,681 left, 6,600 were randomly selected to participate, excluding 145 who were discarded due to data base errors, and 679 who we were unable to trace. On performing household visits, it was apparent that 1389 were not eligible to participate (see Figure 1). This left 4,387 individuals who were potentially eligible. Of these, 363 (8.3%) refused consent. Reasons volunteered for refusal included being too busy to participate.

Interviews were performed on 4,024 individuals including five sets of triplets. From the remaining 4,009 twins there were 1,954 complete twin pairs and 72 unpaired twins. The paired twins comprised 635 all male pairs (364 pairs classified as MZ, 265 as DZ, and 6 unclassified), 785 all female pairs (466 pairs classified as MZ, 311 as DZ and 8 unclassified) and 534 mixed gender pairs. Among the 3980 twin individuals, the mean age was 34.0 years and over three quarters were under 45 years (Table 1), 46.2% were male, and 90.1% were of Sinhalese ethnicity. Over half (53.3%) were married. Among the women 34.6% were employed, as were 72.9% of the men. The mean number of people in twins' households was 4.90. Those who refused had a mean age of 36.2 years, 48.2% were male and 85.7% were of Sinhalese ethnicity. The sex ratio was not significantly different between those who gave consent and those who refused consent, but the refusers were slightly older and slightly less likely to be of Sinhalese ethnicity (Table 2).

**Table 1:** Age distribution of the twin sample (including unpaired twins)

Age group	N	%
15–24	1185*	29.77
25–34	1109	27.86
35–44	790	19.85
45–54	565	14.20
55–64	228	5.73
65–74	81	2.04
75–84	22	0.55

Total	3980	100.00

*Includes two people aged 14 who were not screened out before the interview

**Table 2:** Demographic characteristics: Individuals in the twin sample who consented to participate, compared to those who refused

Characteristic	Consenters N = 3995	Refusers N = 363	Test statistic	*P*
Age (mean)	33.9	36.2	3.12^1^	<0.01
Sex (% male)	46.2	48.2	0.75^2^	0.45
Ethnicity (% Sinhalese)	90.1	85.7	-2.67^2^	0.01

1 *t*-value

2 *z*-value

The mean age for all people over 15 years in the Colombo district of Sri Lanka is approximately 38.7 years, and 51.2% of people are male (calculated from information in the 2001 census [17]).

### Singleton study

Alongside the twins, 2,485 non-twins were randomly selected for interview. Of these 174 were excluded because they have gone abroad or were working far away or could not speak Sinhala, or the interview was of poor quality. Of those selected, 292 (12.6%) refused to participate. A total of 2,019 interviews were completed. The mean age was 43.5 years and 45.6% were male.

## Discussion

The ascertainment of twins for the population based register was thorough and should have included the vast majority of twins actually residing in the area, due to the redundancy of reporting (many reports of twins were duplicates). However, the ascertainment appears to be slightly less thorough in the regions where the election department officers rather than Grama Nildharis administered the questionnaire inquiring about twins [37].

There appear to be no important gender or age differences between the interviewed twin sample and the twins refusing to participate, suggesting the final twin sample is not biased according to selective participation. The mean age of the twins sampled is slightly younger than the mean age for all people 15 years or older in Colombo district. This is likely due to twins in older pairs being more likely to have died or moved far away from one another [18]. Similarly, the twin sample included a slightly higher percentage of females than the average for Colombo district, which may be due to females being less likely to die or move away. The mean age of the singletons sampled was slightly greater than the mean age of all people 15 years or older in Colombo district, which could be due to the singleton sampling method: young adults (roughly age 15–25 years) may have been undersampled because many may still be living at home with their parents until they marry and move away. This would mean that young adults were concentrated in households with several people over age 15, giving them a lower chance of being selected to participate. Also, younger singletons often said they were too busy and less keen to participate in research, but we found that twins of all ages were keen and found time to participate. However, the mean age and sex differences between the twins, singletons and the Colombo census statistics are small so the overall representativeness is good.

This study has contributed to capacity building by translation of psychiatric instruments, including the CIDI, into Sinhalese. A cohort of research workers have been trained in interviewing and general survey research methods, which will enable future epidemiological research. A laboratory will be established for zygosity testing, which will develop the capacity to perform techniques underlying many molecular genetic approaches.

## Conclusion

The Colombo twin register is one of the first population-based twin registers in the developing world. A rigorous ascertainment system has produced a sample capable of exploring the genetic and environmental influences on the variation in many human conditions in this developing country. It could uncover environmental effects that operate in developed countries but have so far been missed due to the narrower range of environments seen in these countries. It will also assess the impact of measured environmental exposures. The focus will initially be on common mental disorders, somatization and substance abuse, but the sample can readily be used to assess other phenotypes.

## Competing interests

The authors declare that they have no competing interests.

## Authors' contributions

SS, HB and MH wrote the paper. SS assisted in the design of the study and was the main study coordinator. He trained and managed the field workers, and was responsible for data collection and quality control; HB cleaned and analysed the data; SNH assisted in coordinating the study, training and managing field workers, and data collection; NG was responsible for training Sri Lankan investigators in the use of the CIDI; YK cleaned and analysed data; DARK was responsible for the molecular and clinical genetics for the study in Sri Lanka, and contributed to the study design; AS is the Principal Investigator in Sri Lanka, and contributed to the establishment of the twin registry, study design, and management of the study; PMcG contributed to the design of the study; MH is Principal Investigator in the UK, with responsibility for the design of the study. With AS he secured funding for the study, coordinated the wider study group, and supervised the local team in practical issues related to study design and data collection. All authors commented on drafts of the paper.

## Pre-publication history

The pre-publication history for this paper can be accessed here:


